# Lysosome-related genes: A new prognostic marker for lung adenocarcinoma

**DOI:** 10.1097/MD.0000000000034844

**Published:** 2023-09-01

**Authors:** Zeyang Hu, Hang Chen, Hongxiang Li, Shuguang Xu, Yinyu Mu, Qiaoling Pan, Jingtao Tong, Guodong Xu

**Affiliations:** a The Affiliated Lihuili Hospital, Health Science Center, Ningbo University, Ningbo, China; b Health Science Center, Ningbo University, Ningbo, China; c Ningbo Medical Center Lihuili Hospital, Ningbo, China.

**Keywords:** immune microenvironment, lung adenocarcinoma, lysosome, network core gene, prognostic signature

## Abstract

Currently, a reliable early prognostic marker has not been identified for lung adenocarcinoma (LUAD), the most common malignancy. Recent studies demonstrated that lysosomal rupture is involved in cancer migration, progression, and immune microenvironment formation. We performed a bioinformatics analysis of lysosomal rupture to investigate whether lysosome-related genes (LRGs) are key in LUAD. The analysis identified 23 LRGs. Cytoscape visualization identified 10 core genes (*CCNA2, DLGAP5, BUB1B, KIF2C, PBK, CDC20, NCAPG, ASPM, KIF4A, ANLN*). With the 23 LRGs, we established a new risk scoring rule to classify patients with LUAD into high- and low-risk groups and verified the accuracy of the risk score by receiver operating characteristic curves and established a nomogram to evaluate clinical patients. Immunotherapy effectiveness between the high- and low-risk groups was evaluated based on the tumor mutational burden and analyses of immune cell infiltration and drug sensitivity. Pathway enrichment analysis revealed that lysosomes were closely associated with glucose metabolism, amino acid metabolism, and the immune response in patients with LUAD. Lysosomes are a likely new therapeutic target and provide new directions and ideas for treating and managing patients with LUAD.

## 1. Introduction

The most recent World Health Organization (WHO) report stated that lung cancer is the second most prevalent malignant tumor and has the highest mortality rate in the world, which seriously affects human health.^[1,2]^ Excluding lung squamous carcinoma, which is closely related to smoking, the incidence rate is currently highest for lung adenocarcinoma (LUAD). Currently, the popularization of low-dose computed tomography and continuous development of personalized early treatment for LUAD has reduced the lung cancer mortality rate somewhat. Therefore, identifying accurate and reliable early prognostic markers is crucial for the survival prognosis of patients with LUAD.

An intracellular organ, the lysosome contains numerous acidic hydrolases, which are an important means of triggering spontaneous apoptosis.^[3,4]^ The contribution of lysosomes, an extremely important part of the cell death process, has been largely overlooked in cancer. The metabolism of cancer cells is extremely fast, and the biological properties of lysosomes meet exactly the needs of tumor growth.^[3,5]^ Therefore, the expression profile of lysosome-related genes (LRGs) is likely to be a relevant condition that predicts early cancer. In recent years, the potential effect of the biological function of lysosomes in tumors has received increasing attention. Soleimani et al induced lysosomal activation by activating TFEB phosphorylation in triple-negative breast cancer, which caused cellular autophagy.^[6]^ Diverse lysosome-mediated cancer therapeutic agents such as strigolactones,^[7]^ tea polysaccharides,^[8]^ and quercetin^[9]^ were identified through the biological properties of lysosomes. Meanwhile, lysosome-induced oxidative burst,^[10]^ histone protease release,^[11]^ and oxidative stress are involved in tumor cell immune escape and apoptosis. Similarly, as an intracellular digestive organ, certain lysosomal metabolic process products are likewise the basis for tumor proliferation and migration. However, the specific mechanisms involved in lysosomal involvement in LUAD and development remain to be explored.

In this study, we developed a new risk scoring system consisting of 23 LRGs to accurately predict the prognosis of patients with LUAD, constructed and validated a nomogram, and explored the response of LRG scores in the immune microenvironment and drug therapy in patients with LUAD. Ten core genes associated with lysosomal-associated LUAD were identified through the protein–protein interaction (PPI) network to determine representative targets for LUAD therapy.

## 2. Materials and Methods

### 2.1. Data preparation and processing

We obtained available follow-up and clinical data for 535 LUAD and 59 normal samples from The Cancer Genome Atlas (TCGA) database as a training cohort. The microarray dataset GSE37745 from the Gene Expression Omnibus (GEO) database was used as a separate cohort for subgroup analysis to verify the data validity. The data were aligned and integrated with Perl software. R software was used for bioinformatics analysis and image plotting. Data were normalized using the R package limma.

### 2.2. LRG acquisition

We collected 877 LRGs from the Gene Ontology Resource (http://geneontology.org/) using the keyword “lysosome.”

### 2.3. Differential analysis between Ensembl annotation and LRGs

We downloaded gene transfer format (GTF) files from Ensembl (http://asia.ensembl.org) to perform gene annotation and ID conversion,^[12]^ which enabled the conversion of gene IDs to gene names to obtain a more accurate gene expression matrix. The differentially expressed LRGs in the intersection of TCGA database and GEO database samples were screened with a false discovery rate < 0.05 and |log2 fold change (FC)| > 0.585. Genes were screened using the R packages limma and pheatmap, Volcano plots and heatmaps were mapped using R gplot and pheatmap.

### 2.4. Calculation of tumor mutational burden and prognostic gene screening

The tumor mutation data were downloaded from TCGA database, integrated by Perl software, and analyzed and visualized by the R package maftools. Univariate Cox regression was used for screening and forest diagrams of prognostic genes, with *P* < .05 as the screening criterion. The Wilcoxon rank test was used for the risk ratio (RR) and 95% confidence interval (CI), which were run by the R packages survminer and survival.

### 2.5. Construction and validation of LRG prognostic risk scoring model

The prognostic genes with *P* < .05 in the training group were identified using univariate Cox regression analysis. A LASSO regression model was constructed to identify the genes with the least error in cross-validation,^[13]^ and the genes involved in the model construction were derived. The risk score formula was obtained as follows: $LRG\;risk\;score=\underset{i}{\overset{n}{\sum }}\;Coef\;f\;icient\left(gene\right)*Expression\left(gene\right)$. The training group was divided into high- and low-risk groups according to the risk score, and the median was used as the critical value. Principal component analysis (PCA) was performed on the model plots and the PCA plots were plotted using the R package ggplot2. The R packages survival and survminer were used for survival analysis and progression-free survival analysis and plotting.

### 2.6. Independent prognostic analysis and correlation analysis of clinical characteristics

Using the sample age, sex, pathological stage, T classification, and risk score as variables, univariate and multivariate Cox independent prognostic analyses were performed at *P* < .05 to obtain the corresponding hazard ratio (HR) values and their fluctuation ranges.

### 2.7. Immunotyping analysis

We classified patients with LUAD into 5 subtypes using the file Subtype_Immune_Model_Based.txt for pan-cancer immunophenotyping and observed whether the patients’ risk scores differed between subtypes with the R package ggpubr.

### 2.8. Nomogram analysis

A nomogram of the corresponding variables was plotted with the R packages survival and regplot to assess the clinical prognoses of the corresponding patients, and 1-, 3-, and 5-year calibration curves were plotted. Receiver operating characteristic (ROC) curves were plotted with the R package timeROC to compare the risk score validity for predicting the patients’ clinical risk. Univariate and multifactorial Cox risk regression prognostic analyses were performed on the nomogram.

### 2.9. Immunocyte correlation analysis

Tumor-infiltrating immune cells were identified from tumor sequencing data using the ESTIMATE (Estimation of STromal and Immune cells in Malignant Tumor tissues using Expression data) algorithm. Subsequently, the R packages reshape2 and ggpubr were used to analyze the differences in the infiltration of different immune cells between the high- and low-risk groups and to draw boxplots. Single-sample gene set enrichment analysis (ssGSEA) and gene set variation analysis (GSVA) were performed using the R packages GSEABase and GSVA, respectively, and yielded a boxplot of immune-related functions and a heat map of differential immune-related functional pathways. The Tumor Immune Dysfunction and Exclusion (TIDE) score file for LUAD was downloaded from the TIDE database (http://tide.dfci.harvard.edu/) to assess anti-immune checkpoint therapy efficacy.

### 2.10. Gene mutation and drug sensitivity analyses

Box plots for gene mutation and drug sensitivity analyses were created with the R packages ggpubr and pRRophetic.

### 2.11. PPI network and network core genes

The PPI network of differential genes was constructed and visualized in string-db.org/ and the core network genes were extracted to assess the differential expression of the core genes in the training cohort.

### 2.12. Statistical analysis

Differences between multiple groups were analyzed with the Kruskal–Wallis test. Differences between 2 groups were compared with the Wilcoxon test. *P* < .05 was considered statistically significant. R 4.2.1 and Strawberry Perl software were used for all statistical analyses.

## 3. Results

### 3.1. Expression analysis of LRGs in patients with LUAD

**Figure 1.**
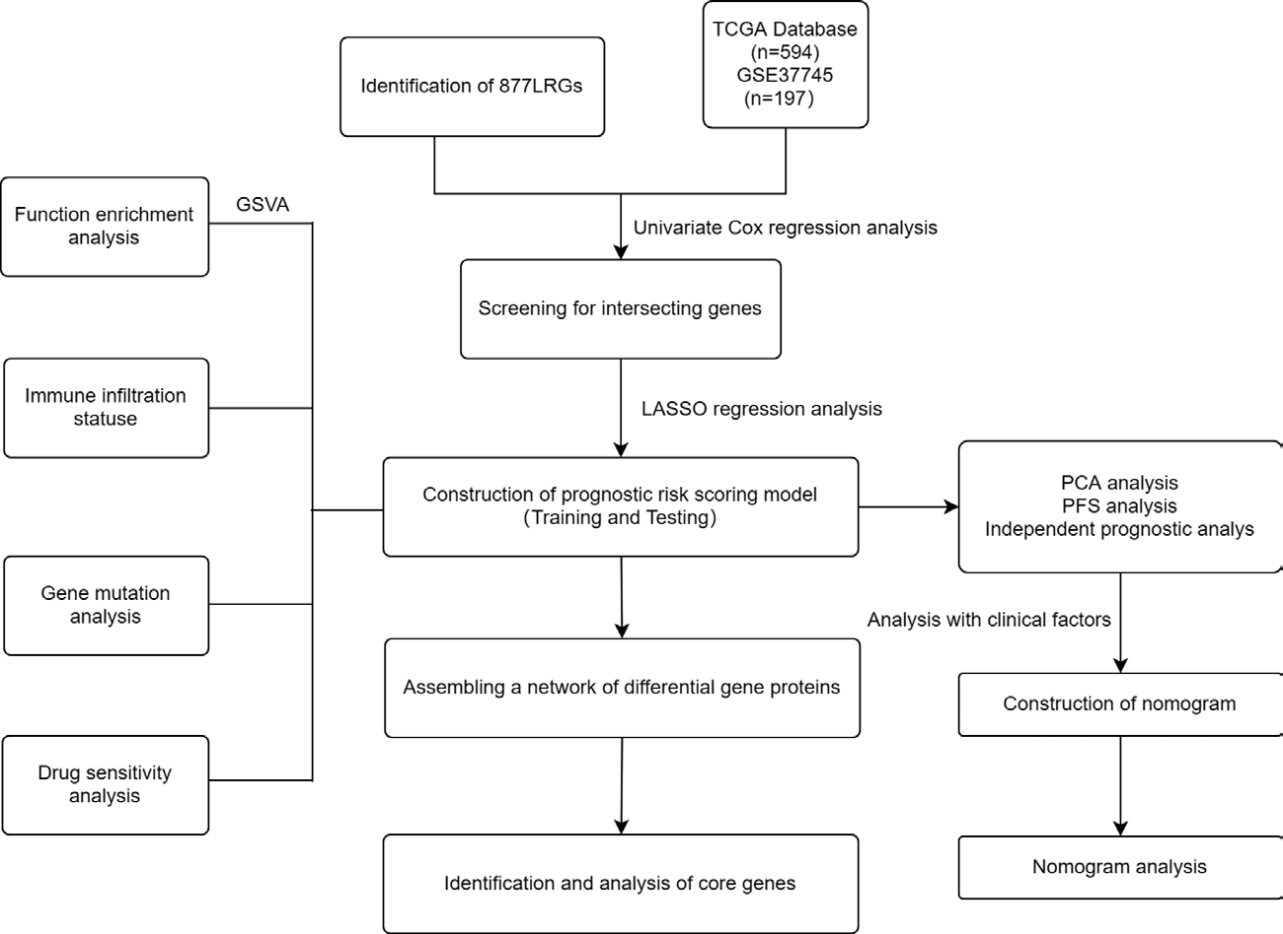
Flow diagram of study design. GSVA = gene set variation analysis, LRGs = lysosome-related genes, PCA = principal component analysis, PFS = progression-free survival, TCGA = The Cancer Genome Atlas.

**Figure 2.**
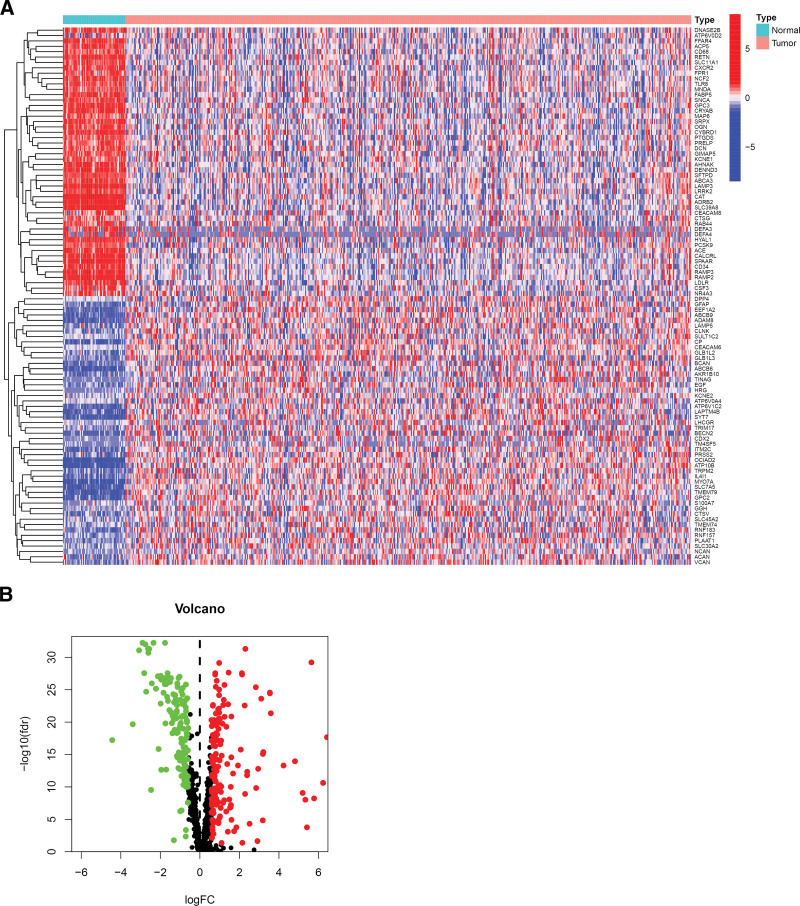
Expression analysis of LRGs in LUAD. (A) Heat map (red represents upregulated genes, blue represents downregulated genes). (B) Volcano plot (red dots represent upregulated genes, green dots represent downregulated genes, black dots represent genes without differences). LRGs = lysosome-related genes, LUAD = lung adenocarcinoma.

Figure 1 depicts the flow chart of the processes used in this research. We included 523 patients and 59 normal samples to investigate the effect of LRGs on patients with LUAD and identified 877 LRGs. Of these, 291 differential LRGs were extracted by comparing the differences between tumor and normal tissues, where 152 genes were downregulated and 139 genes were upregulated (Fig. 2A and B).

### 3.2. Construction of prognosis-related LRGs

**Figure 3.**
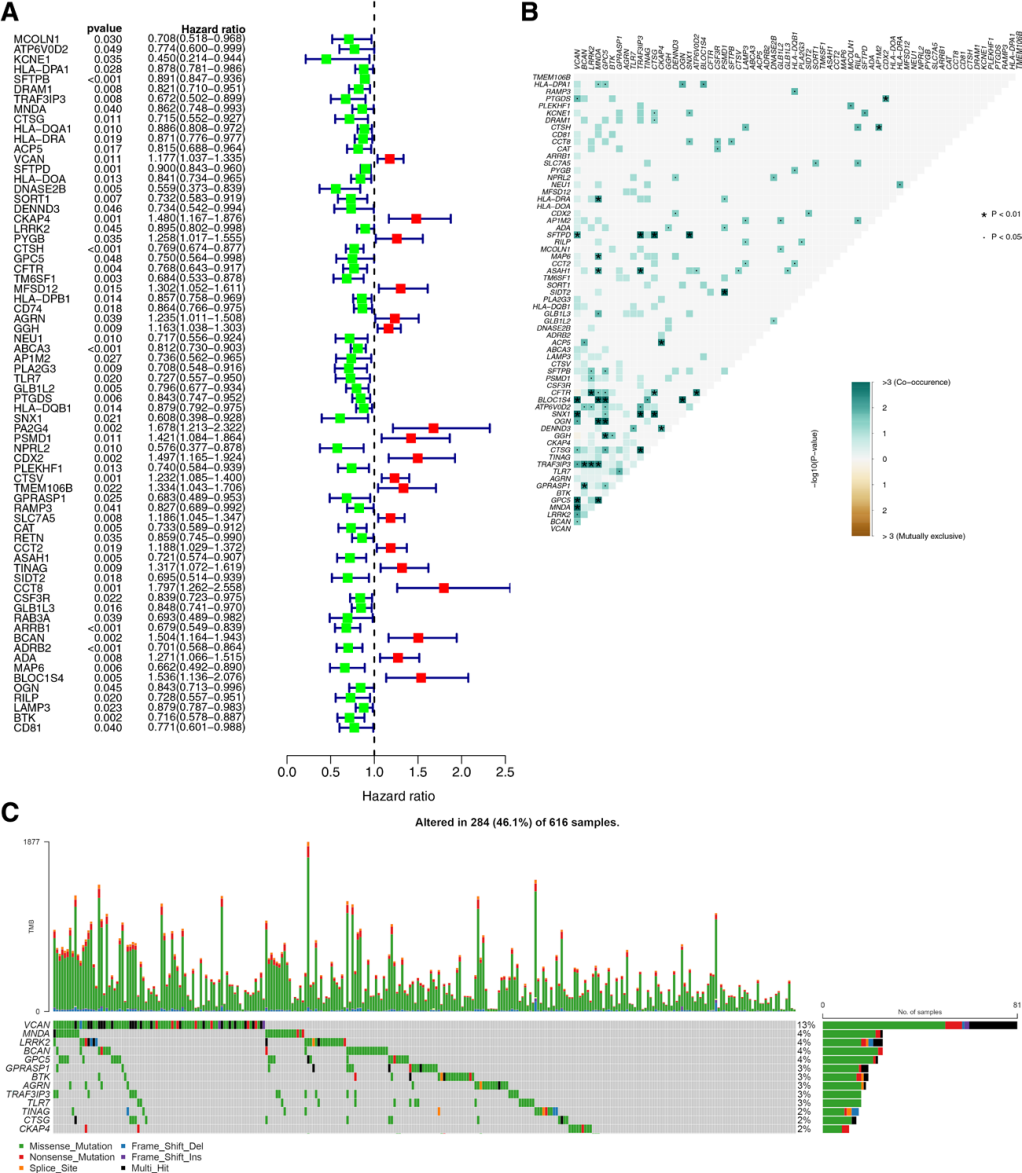
LRG differential analysis and mutation analysis in LUAD. (A) Forest plot of differential genes (red represents upregulated genes, blue represents downregulated genes). (B and C) Mutation frequencies and co-mutation relationships of LRGs. LRGs = lysosome-related genes, LUAD = lung adenocarcinoma.

Seventy LRGs were associated with LUAD prognosis, as shown in the forest plot in Figure 3A. A total of 284 samples (46.1%) were mutated, with the *VCAN* gene demonstrating the highest mutation rate of 13%. Most of the mutations were missense mutations. In descending order of mutation rate, the remaining mutated genes were *MNDA, LRRK2, BCAN*, and *GPC5* (Fig. 3C). The co-mutation relationship between the genes is depicted in Figure 3B.

### 3.3. Prognostic model construction and correlation analysis

**Figure 4.**
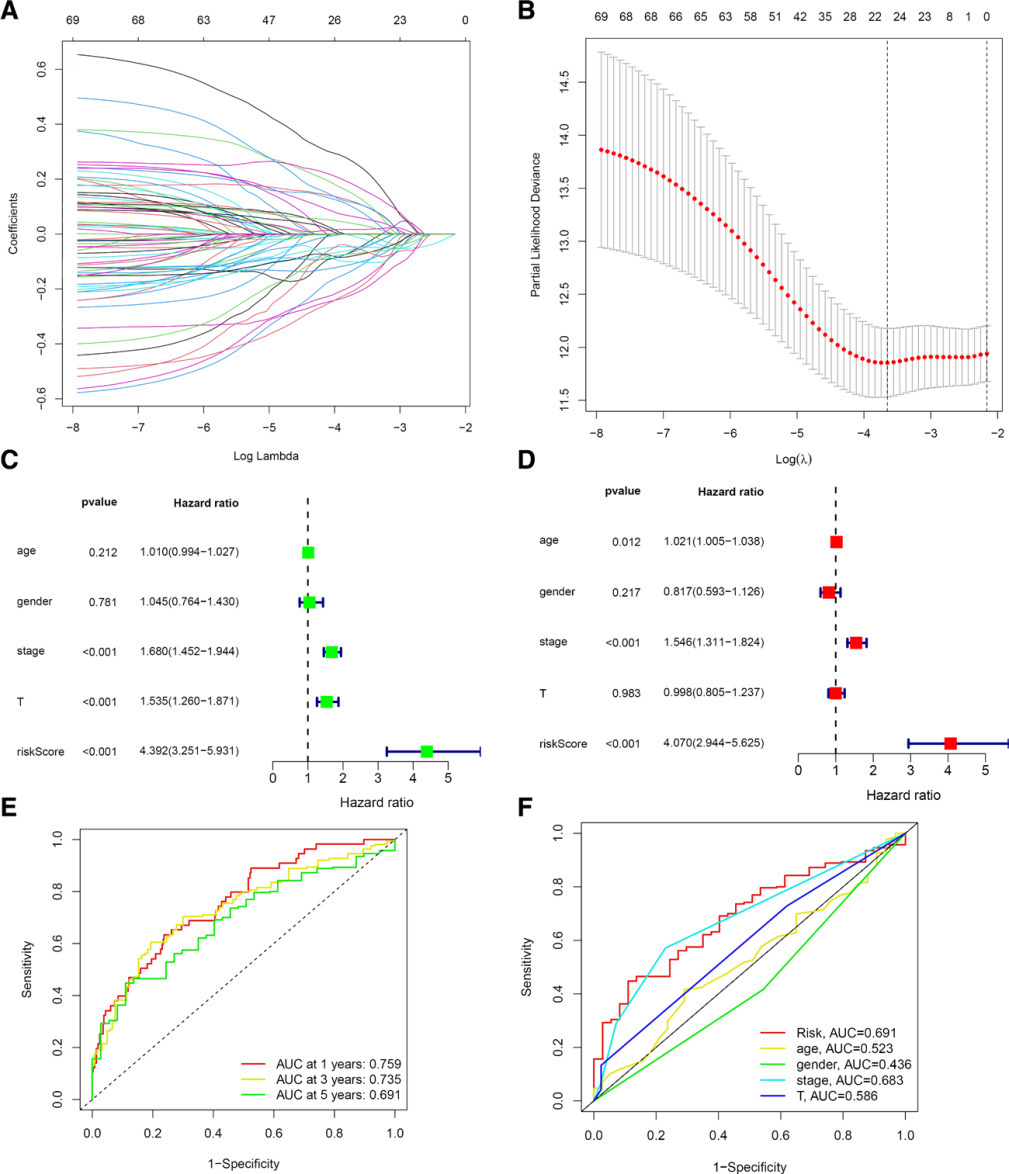
Construction of a lysosome-associated prognostic gene signature. (A and B) The coefficient and partial likelihood deviance of the prognostic signature. (C and D) Univariate and multivariate risk proportion regressions demonstrated that the risk score was significantly associated with prognosis. (E and F) ROC curves demonstrate a greater predictive power of risk scores. ROC = receiver operating characteristic.

The risk score model was constructed with TCGA database samples as the training group and GEO database samples as the test group, where the samples were divided into high- and low-risk groups (Fig. 4A and B). Furthermore, we screened 22 LRGs involved in the model construction, and the specific risk scores were calculated as follows: LRG risk score = *SFTPB* × −0.04678 + *TRAF3IP3* × −0.05291 + *VCAN* × 0.06091 + *SORT1* × −0.10461 + *PYGB* × 0.11242 + *GPC5* × −0.12215 + *TM6SF1* × −0.17787 + *AGRN* × 0.08867 + *NEU1* × −0.21211 + *AP1M2* × −0.19820 + *GLB1L2* × −0.05107 + *NPRL2* × −0.101839922026203 + *CDX2* × 0.01698 + *PLEKHF1* × −0.07628 + *TMEM106B* × 0.13589 + *CCT2* × 0.03545 + *CCT8* × 0.25253 + *RAB3A* × −0.05902 + *ARRB1* × −0.01176 + *BCAN* × 0.17272 + *MAP6* × −0.07034 + *BLOC1S4* × 0.10990 + *BTK* × −0.05234.

**Figure 5.**
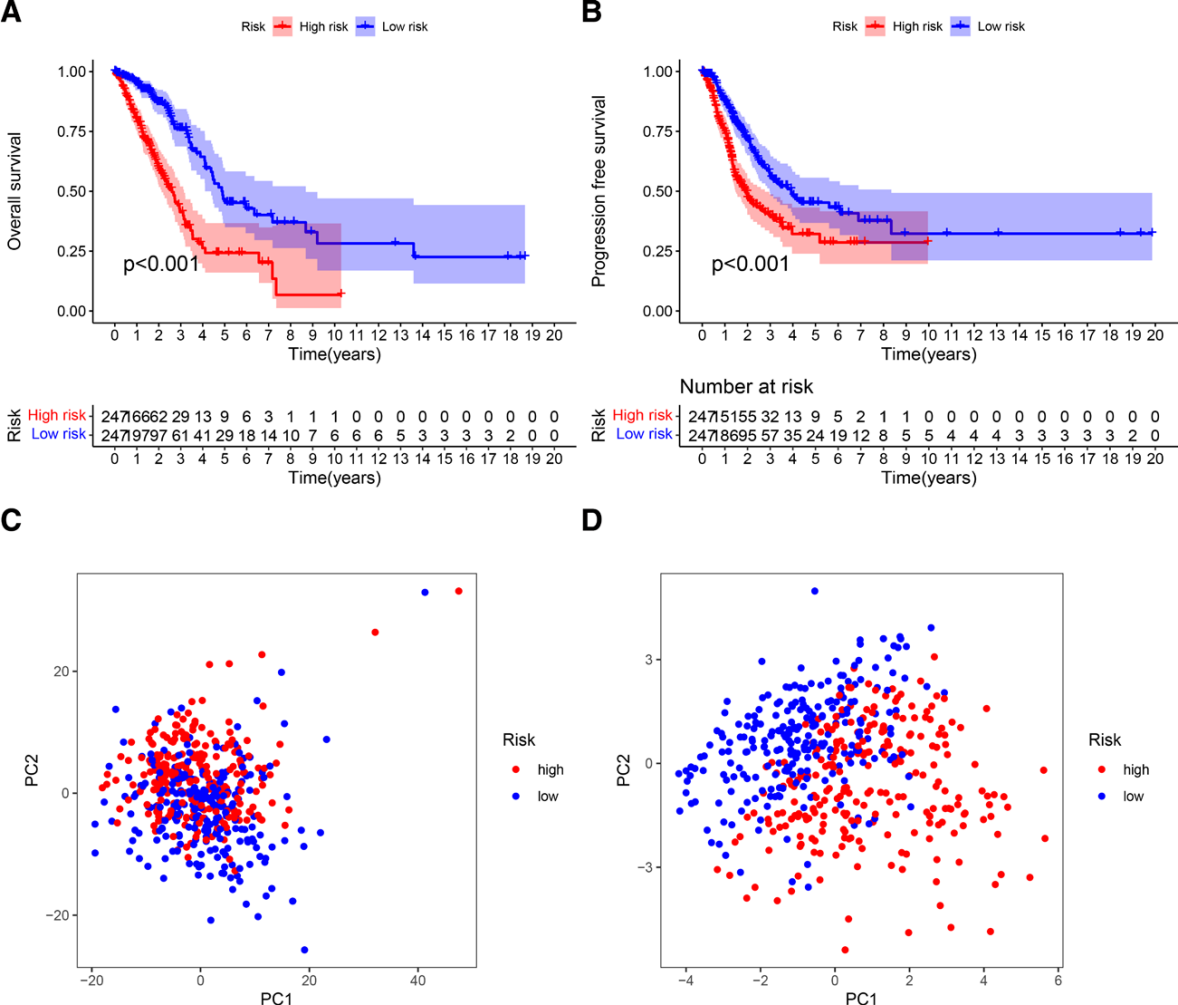
(A and B) Validation of OS and PFS. (C and D) PCA verification of training group and our model. PCA = principal component analysis, PFS = progression-free survival.

The validity of the risk score was subsequently assessed by univariate and multivariate Cox risk regressions, which determined that the risk score could be used as an independent risk factor as compared with other clinical factors (univariate Cox analysis: HR = 4.392; 95% CI: 3.251–5.931; multifactorial Cox analysis: HR = 4.070; 95% CI: 2.944–5.625) (Fig. 4C and D). Figure 4F demonstrates that the area under the curve (AUC) value of the risk score ROC curve was greater than that of the other clinical characteristics (age, sex, stage, T), indicating that our model had higher accuracy for prognostic rather than clinical assessment of patients. The AUC values were 0.759, 0.735, and 0.691 for patients at 1, 3, and 5 years postoperatively (Fig. 4E), respectively, again demonstrating the excellent prognostic value assessment of our model. Subsequently, survival analysis was performed for the 2 groups, where Kaplan–Meier analysis demonstrated that patients with LUAD with higher LRGs scores had poorer survival curves (Fig. 5A). The PCA demonstrated that we were able to distinguish better between the high- and low-risk groups (Fig. 5C and D). Furthermore, the progression-free survival proved that the low-risk group had a better prognosis than the high-risk group (Fig. 5B).

### 3.4. Nomogram analysis

**Figure 6.**
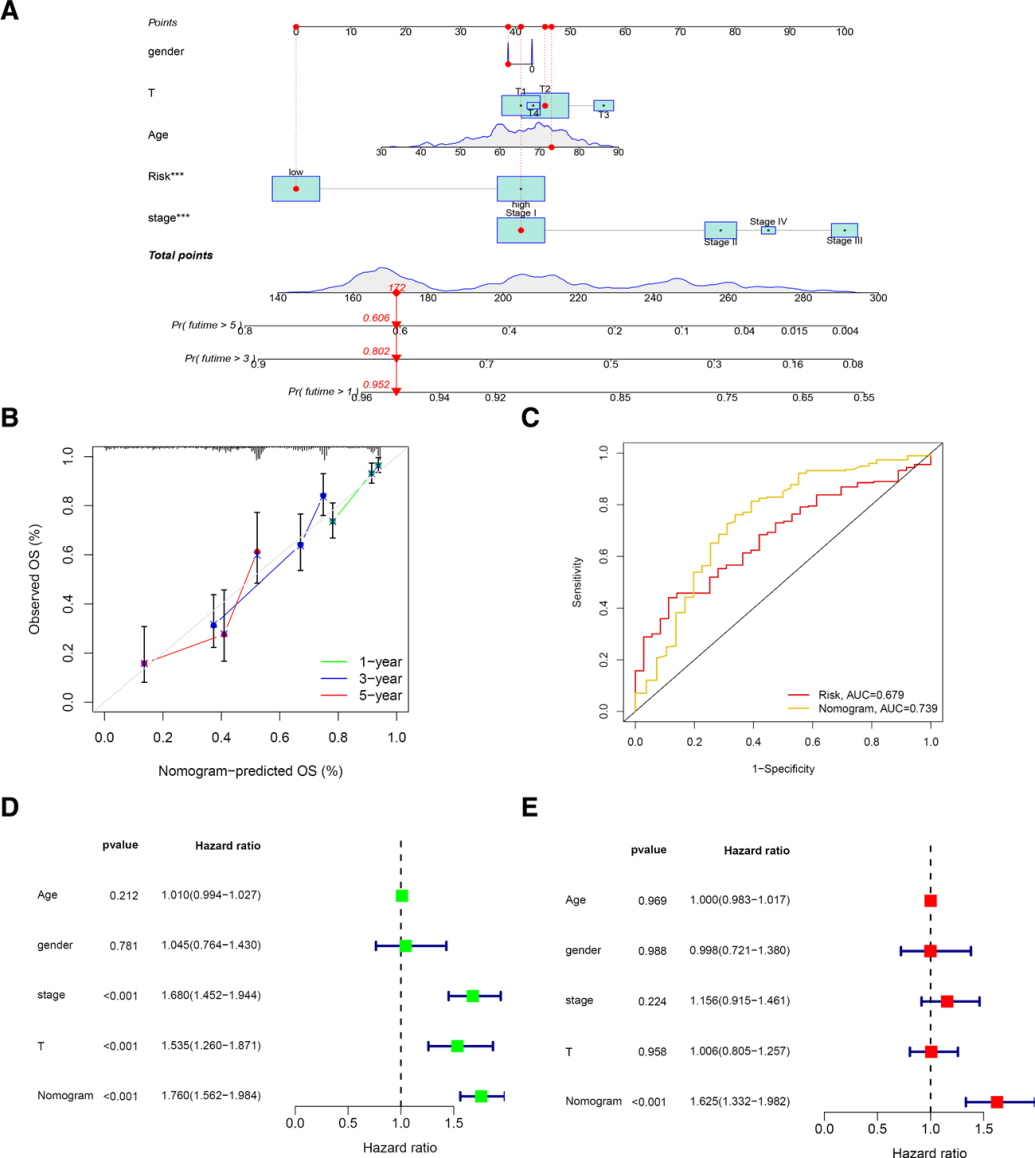
Nomogram analysis. (A) Nomogram. (B) Nomogram stability. (C) Nomogram ROC curve. (D and E) Univariate and multivariate risk proportion regressions demonstrate that the nomogram was significantly associated with prognosis. ROC = receiver operating characteristic.

By plotting the nomogram related to sex, age, pathological stage, T stage, and risk score, we were able to obtain the scoring of the corresponding patients and thereby assess the prognostic risk by the calculated total score (Fig. 6A) and predict the 1-, 3-, and 5-year survival rates of the patients with LUAD. Figure 6B demonstrates the stability of the nomogram. By comparing the ROC curves of the nomogram and the LRG risk score, we determined that the nomogram had a greater AUC value than the LRG risk score (Fig. 6C). Finally, we analyzed whether the nomogram could be an independent factor for patients with LUAD with LRGs through univariate and multivariate Cox risk regressions, and the results were statistically significant.

### 3.5. Correlation analysis of immune function

**Figure 7.**
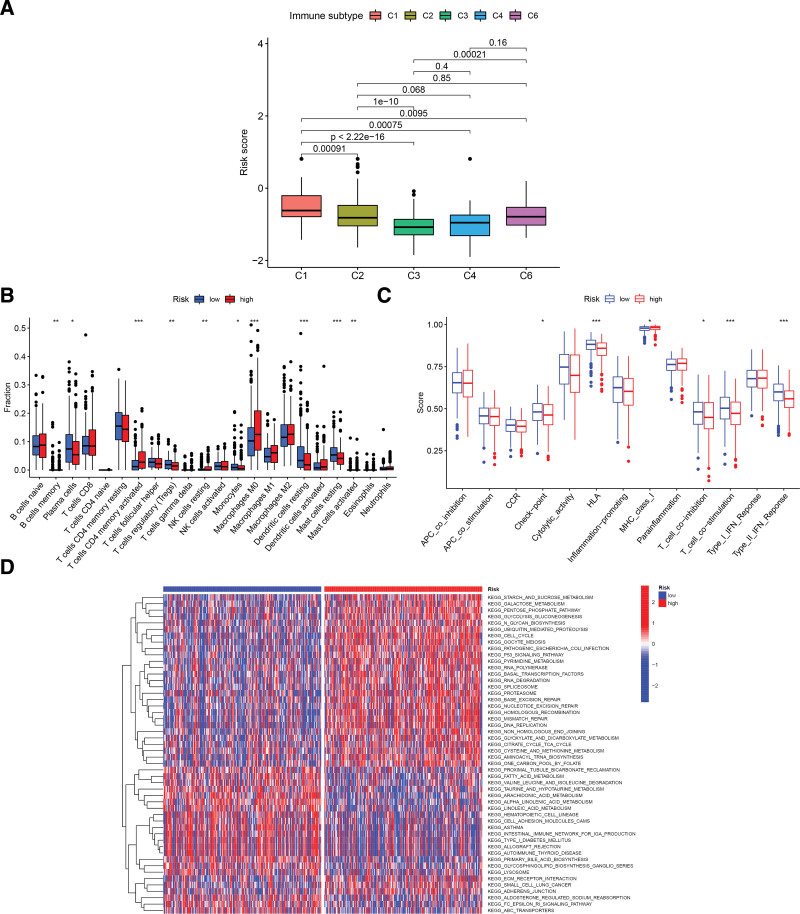
(A) Immunotyping analysis. (B) Expression of immune cells in different risk groups. (C) Different cell functions in different risk groups. (D) GSVA. *P < .05; **P < .01; ***P < .001. GSVA = gene set variation analysis.

First, we observed that, between the different pan-cancer immunophenotypes, C1 patients differed from all other phenotypes and had higher risk scores than patients of the other phenotypes (Fig. 7A). Second, by assessing paracancer immune cell infiltration, we were able to determine that the low-risk group had a significantly higher abundance of memory B-lymphocytes, plasma cells, regulatory T cells, monocytes, resting dendritic cells, and resting mast cells than the high-risk group, while the tumor tissue from the high-risk samples contained significantly more infiltrated activated CD4 memory T cells, resting natural killer cells, M0 macrophages, and activated mast cells (Fig. 7B). The low-risk group had higher scores for immune checkpoint, human leukocyte antigen, T-cell co-stimulation, T-cell co-inhibition, and type II IFN response infiltration than the high-risk group (Fig. 7C). Finally, GSVA determined that glucose and nuclear metabolism and the P53 signaling pathway were enhanced in the high-risk group, while amino acid and fatty acid metabolism were more pronounced in the low-risk group (Fig. 7D).

### 3.6. Analysis of gene mutations and drug efficacy

**Figure 8.**
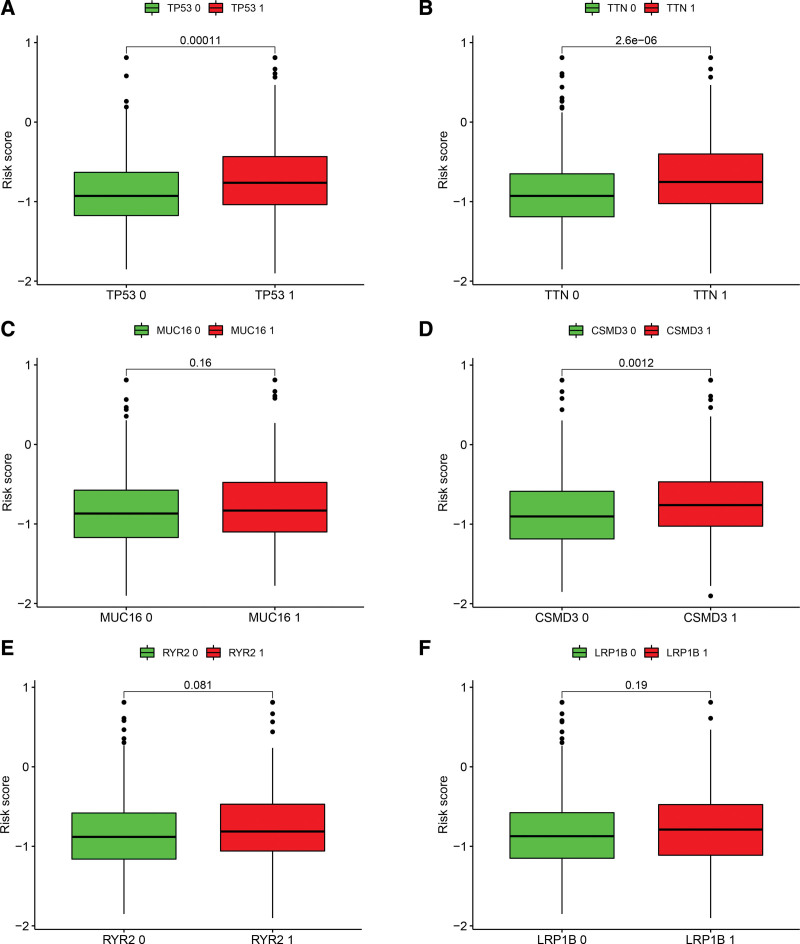
Mutation analysis (0: wild-type; 1: mutant).

**Figure 9.**
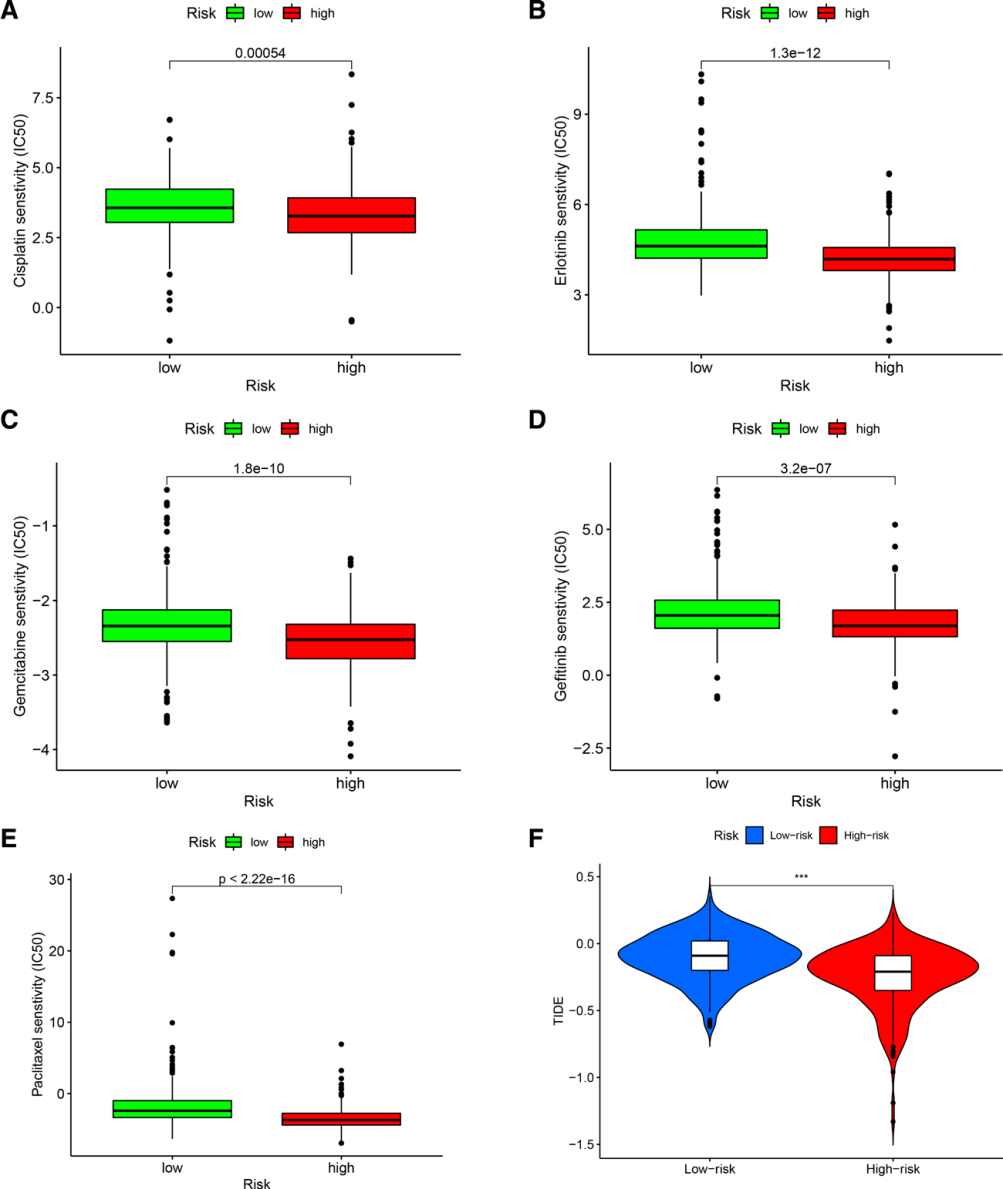
Analysis of drugs and TIDE with risk score. TIDE = The Tumor Immune Dysfunction and Exclusion.

Among the 6 genes with a high proportion of mutations, the mutant phenotypes of *TP53, TTN*, and *CSMD3* had significantly higher risk scores than the wild-type (Fig. 8A–F). Drug sensitivity analysis determined that cisplatin, erlotinib, gefitinib, gemcitabine, and paclitaxel had higher effectiveness in the low-risk group than in the high-risk group, and TIDE scores were negatively associated with the risk score (Fig. 9A–F).

### 3.7. LRG functional enrichment analysis

**Figure 10.**
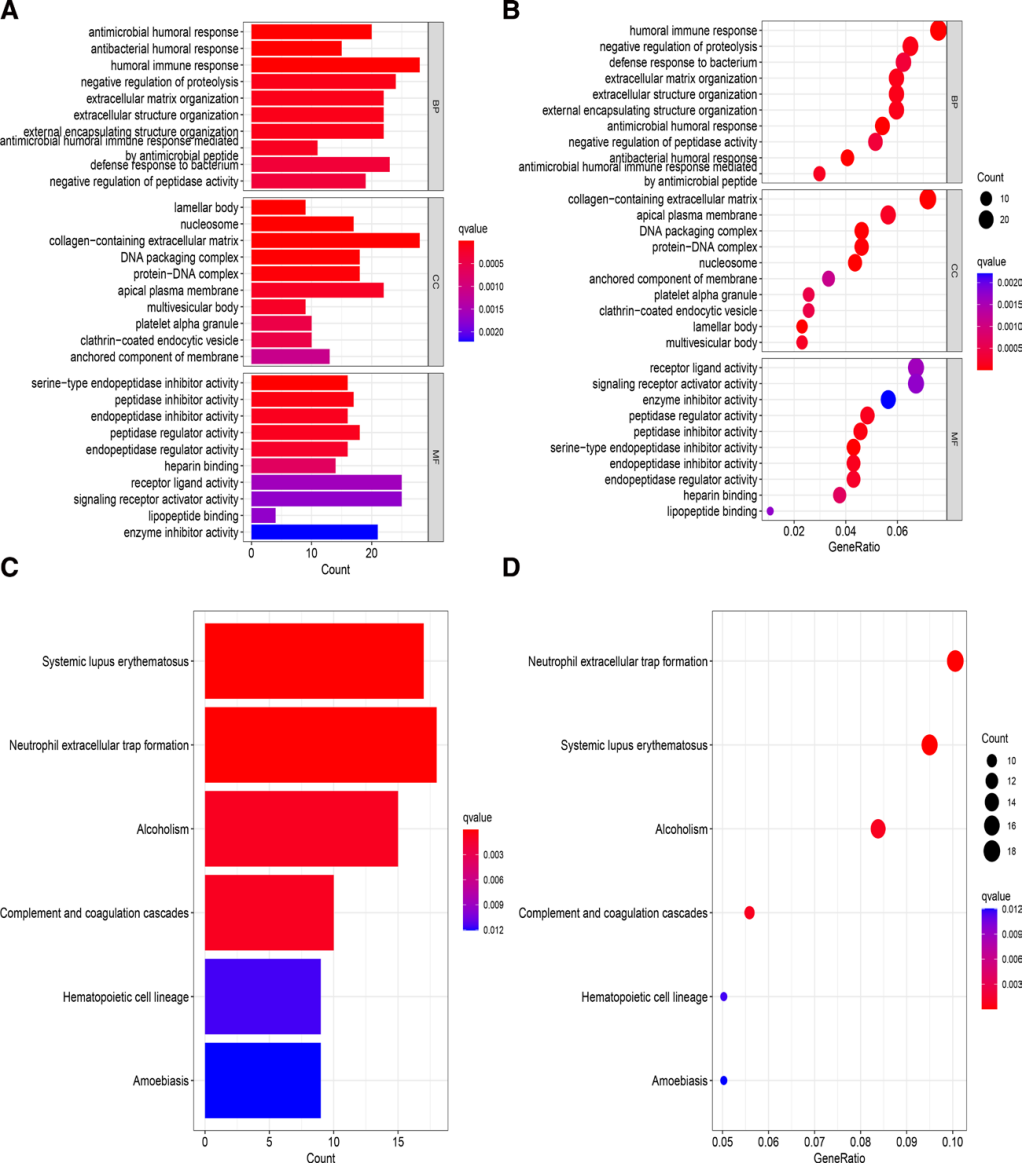
GO and KEGG analyses of LRGs in LUAD. GO = Gene Ontology, KEGG = Kyoto Encyclopedia of Genes and Genomes, LRGs = lysosome-related genes, LUAD = lung adenocarcinoma.

We performed Gene Ontology (GO) and Kyoto Encyclopedia of Genes and Genomes (KEGG) pathway enrichment analysis to understand the role of prognostic markers in LUAD. GO analysis demonstrated that prognostic indicators were more abundant in human immune responses such as antimicrobial humoral immune response mediated, defense response to bacterium, and antimicrobial humoral response (Fig. 10A and B). KEGG analysis demonstrated that the most abundant pathways for LRGs were neutrophil extracellular trap formation and others (Fig. 10C and D).

### 3.8. Network core genetic analysis

**Figure 11.**
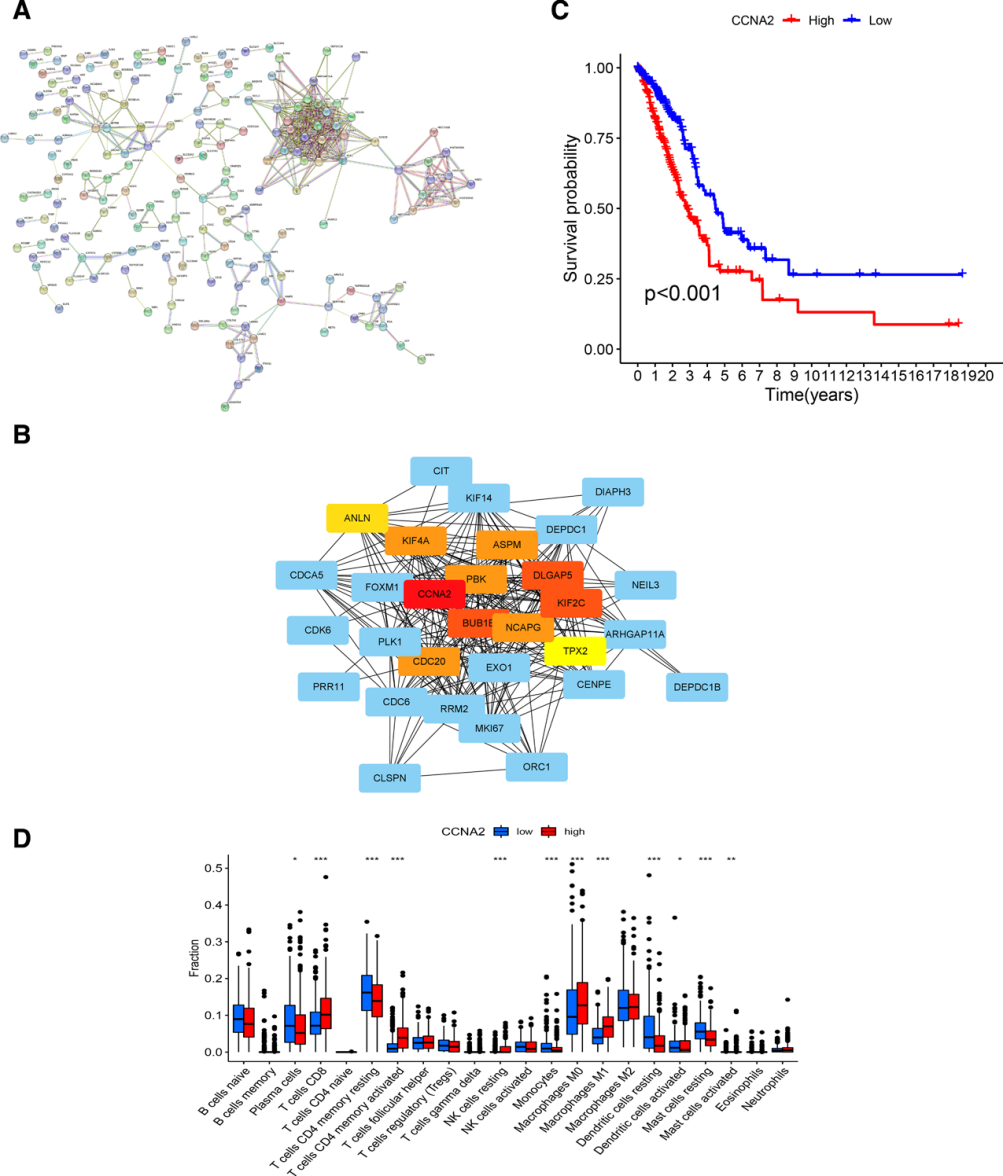
Network core genes. (A) PPI network. (B) Visualization of network core genes. (C, D) OS and immune infiltration analysis of *CCNA2*. PPI = protein–protein interaction.

We constructed a PPI network between LRGs to investigate the interactions between LRGs (Fig. 11A), in which the nodes represented genes or proteins and a linkage between 2 nodes indicated a connection between 2 proteins. Figure 11B depicts the extracted 10 core protein interaction genes (*CCNA2, DLGAP5, BUB1B, KIF2C, PBK, CDC20, NCAPG, ASPM, KIF4A, ANLN*), where the red area indicates a core gene of protein interactions. Samples with higher expression of the core gene *CCNA2* rather than low expression had worse prognoses (Fig. 11C). Plasma cells, resting CD4 memory T cells, monocytes, dendritic cells, and mast cells were more abundant in tumors with low *CCNA2* expression, and there was greater infiltration of CD8 T cells, activated CD4 memory T cells, resting natural killer cells, and M0 macrophages in samples with high *CCNA2* expression (Fig. 11D). Figures S1–S3, Supplemental Digital Content, http://links.lww.com/MD/J561 depict the survival curves and immune cell differential analysis of the other 9 core genes.

## 4. Discussion

One of the prevalent organelles in the cell, the lysosome contains diverse hydrolytic enzymes and is often used to separate substances that enter the cell from the outside and to digest local cytoplasm or organelles, such as proteins recognized by HSC70.^[14]^ The lysosome also cleaves when the cell turns senescent, thereby digesting the entire cell and causing its death. Recent studies suggested that the lysosome may be essential in lung cancer development and apoptosis,^[15]^ although the potential molecular mechanism of the lysosome as a member involved in apoptosis and its related genes in LUAD have not been reported. We believe that LRGs may play a greater role in LUAD development. Therefore, we constructed an LRG-associated clinical model of LUAD and identified 23 lysosome-associated genetic markers. To clarify the significance of LRGs in LUAD development, we investigated and validated the prognostic value of LRGs in LUAD by constructing a clinical risk score for LUAD-associated LRGs.

We first developed an LRG-associated LUAD model by Cox risk regression, and the prognosis-related model was associated with 23 genes (*SFTPB, TRAF3IP3, VCAN, SORT1, PYGB, GPC5, TM6SF1, AGRN, NEU1, AP1M2, GLB1L2, NPRL2, CDX2, PLEKHF1, TMEM106B, CCT2, CCT8, RAB3A, ARRB1, BCAN, MAP6, BLOC1S4, BTK*), some of which may serve as LUAD biomarkers. For example *VCAN* was associated with the proliferation and migration of a variety of cancers^[16–18]^ while BTK, a tyrosine kinase, was also strongly associated with hematologic tumors.^[19]^ We conjectured that these 23 LRGs may be involved in the progression and metastasis of LUAD. Newman et al^[20]^ found that TRAF3IP3 can upregulate the TGF-β signaling pathway, promote cellular autophagy, and activate CD40 to activate the immune response by promoting NFκB activated.^[21,22]^ Similarly, TM6SF1, NEU1, NPRL2, and TMEM106B, as key genes for lysosome formation, could enhance lysosomal function and thus further regulate tumor progression.^[23,24]^ Epithelial mesenchymal transition (EMT), a crucial stage in building the tumor microenvironment, plays an integral part in the progression of LUAD. Yuan et al found that Glypican-5, an oncogene, could inhibit the process of EMT in lung cancer, so as to suppress tumor growth and metastasis.^[25,26]^ In contrast, PYGB, NEU1, AGRN, ARRB1, MAP6, CDX2, and other genes have been shown to promote EMT and thus enhance LUAD proliferation and migration via the WNT pathway^[27–29]^ or PI3K/Atk pathway.^[30,31]^ A major component of the extracellular matrix, VCAN has also been much studied in recent years, mainly involved in cell adhesion, proliferation, migration and angiogenesis, and as a key mediator of immunity and inflammation to promote the synthesis and secretion of inflammatory factors (TNFα, IL6, NF-κB, etc.).^[32]^ Consequently, these 23 lysosome-related genes affect lysosome construction, EMT, surrounding immune microenvironment and inflammation, and are closely related to the development of LUAD. We also hope that these 23 LRGs can be used as reliable early prognostic markers for lung adenocarcinoma. In our model, the patients with LUAD were divided into high- and low-risk groups according to their risk scores and we confirmed that the risk score level correlated with the patients’ prognoses.

The immune function analysis revealed that the low-risk group had much greater dendritic cell abundance than the high-risk group, which was consistent with the report by Iulianna et al^[15]^ GSVA revealed that cellular pathways such as the P53, carbohydrate metabolism, amino acid metabolism, and nucleotide metabolism pathways were significantly enhanced in the high-risk group, and that the P53 signaling pathway could induce lysosomal rupture and therefore apoptosis.^[33,34]^ Mitsuhiro Endoh demonstrated that FLCN inhibited lysosomal activity through TFE3 to prevent excessive glycoisomerization.^[35]^ We also performed GSEA, and GO and KEGG analysis revealed that the pathways for defense response to bacterium, humoral immune response, and antibacterial humoral response were enriched mainly in the high-risk group, suggesting that lysosomes are associated with in vivo immunity. The immune-related factors were also closely associated with LUAD development.

We plotted a nomogram to assess the survival prognosis of clinical patients. Figure 6A demonstrates that the patients’ prognoses were closely related to the risk score of our constructed model and the tumor pathological stage (*P* < .001), which was used to accurately predict the 1-, 3-, and 5-year survival time of LUAD. In the ROC curve, an AUC value of 0.739 was obtained for the column line graph, which was significantly higher than the other factors. Additionally, we determined that the nomogram and prognostic risk score were independent prognostic factors following the integration of common clinical features (Figs. 4, 6D and 6E).

Notably, a recent study reported that immunotherapy achieved relatively positive results in patients with LUAD.^[36,37]^ Accordingly, we performed immune correlation studies on this model and included analyses of immune cell infiltration, immunophenotyping, and drug sensitivity. In particular, the immune microenvironment is an important aspect in cancer development,^[38]^ where we identified significantly higher infiltration of B cells, T cells, and dendritic cells in the low-risk group, suggesting that a decrease in these cells may be associated with poor prognosis. As first-line therapeutic agents for non-small cell lung cancer,^[39,40]^ cisplatin and paclitaxel significantly improve the survival prognosis of patients with LUAD. In this study, we determined that the low-risk group had significantly higher cisplatin and paclitaxel sensitivity than the high-risk group, indicating that the efficacy of these drugs was significantly higher in the low-risk group than in the high-risk group.

Boyle et al indicated that complex disease traits are driven by numerous small effects, and disease risk is likely to result from the propagation of a network built by regulating several genes.^[41]^ The core genes in this network are likely to drive the surrounding genes to play a crucial role in disease development. Therefore, we performed Cytoscape visualization analysis through the PPI network to identify 10 core genes (*CCNA2, DLGAP5, BUB1B, KIF2C, PBK, CDC20, NCAPG, ASPM, KIF4A, ANLN*) and determined that abnormal expression of these 10 genes was closely associated with LUAD.

Our study has some limitations. First, TCGA and GEO databases were not representative of the clinical situation. Second, we were unable to add detailed experimental studies such as validation analysis using cell lines, animal models, and a large number of clinical samples. In addition, our constructed models required numerous clinical samples for immunohistochemical analysis to verify the prognostic marker validity. Future studies can explore the specific mechanism of action of these genes in lung cancer and thereby select representative targets for LUAD treatment.

## 5. Conclusion

We performed a comprehensive bioinformatics analysis of LRGs in LUAD, established a model with 23 associated genes, and identified the early diagnostic and prognostic features of LRGs in LUAD. Subsequently, we analyzed the risk score, immune cell infiltration, gene mutations, drug sensitivity, pathway enrichment, and network core genes to validate the high practical value of our model and provide new targets and perspectives for LUAD treatment.

## Acknowledgments

We thank the TCGA database and GEO database for generously sharing a large amount of data.

## Author contributions

**Conceptualization:** Zeyang Hu, Yinyu Mu.

**Data curation:** Zeyang Hu, Qiaoling Pan, Guodong Xu.

**Formal analysis:** Hang Chen, Qiaoling Pan, Guodong Xu.

**Funding acquisition:** Hang Chen, Guodong Xu.

**Investigation:** Hongxiang Li.

**Methodology:** Hongxiang Li, Jingtao Tong.

**Project administration:** Shuguang Xu.

**Resources:** Zeyang Hu, Shuguang Xu.

**Software:** Zeyang Hu, Yinyu Mu.

**Supervision:** Zeyang Hu, Yinyu Mu.

**Validation:** Zeyang Hu, Guodong Xu.

**Visualization:** Zeyang Hu, Guodong Xu.

**Writing – original draft:** Zeyang Hu.

**Writing – review & editing:** Zeyang Hu, Guodong Xu.

## Supplementary Material
