# Supplementary material for: Lysosome-related genes: A new prognostic marker for lung adenocarcinoma
Source: Medicine (Baltimore). 2023 Sep 1;102(35):e34844. doi: 10.1097/MD.0000000000034844 (PMC10476855; doi:10.1097/MD.0000000000034844)

Supplementary Materials

Supplementary Figures 1, 2, and 3：OS and immune infiltration analysis of other nine core genes.

Supplementary Figure 1

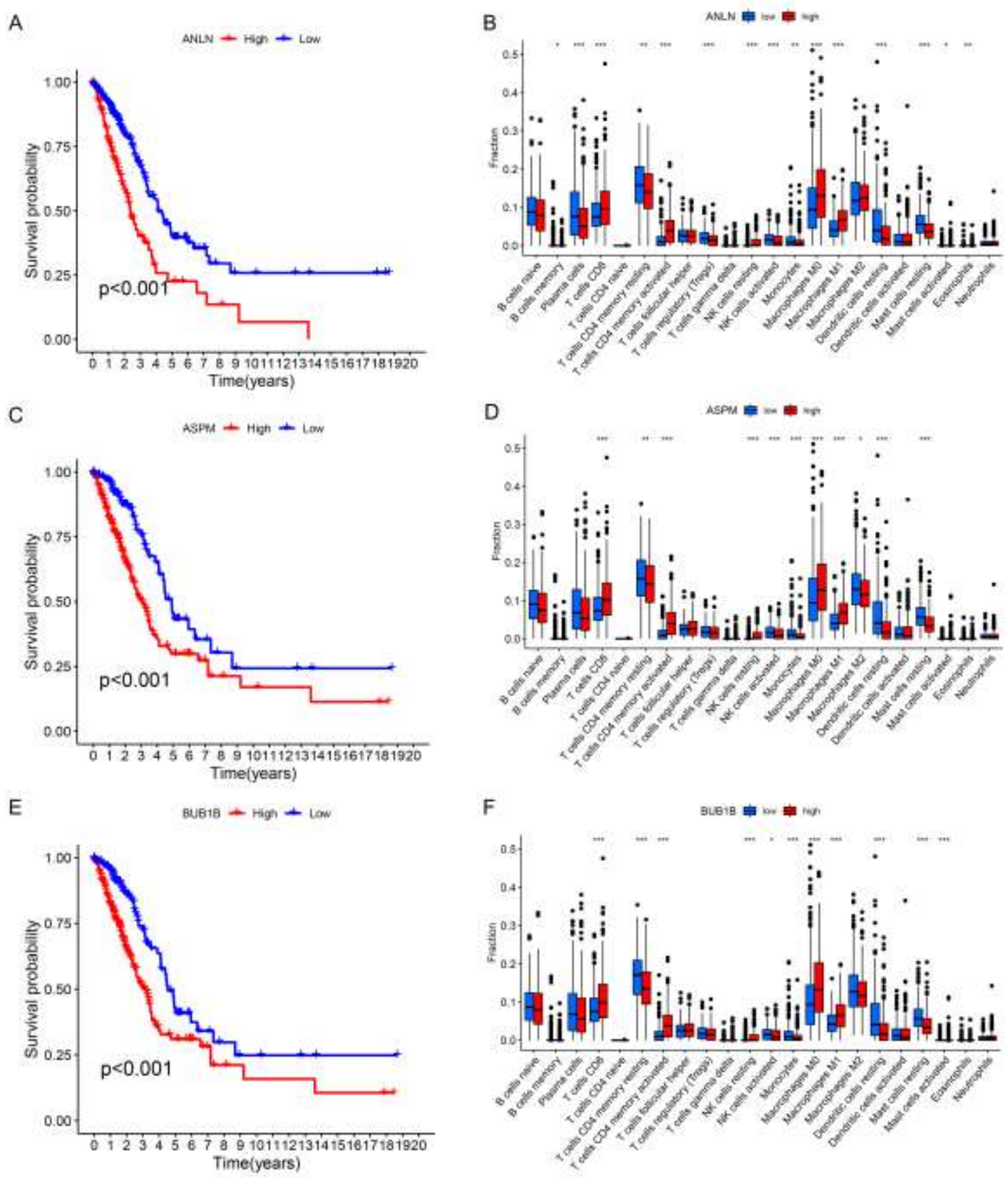

Supplementary Figure 2

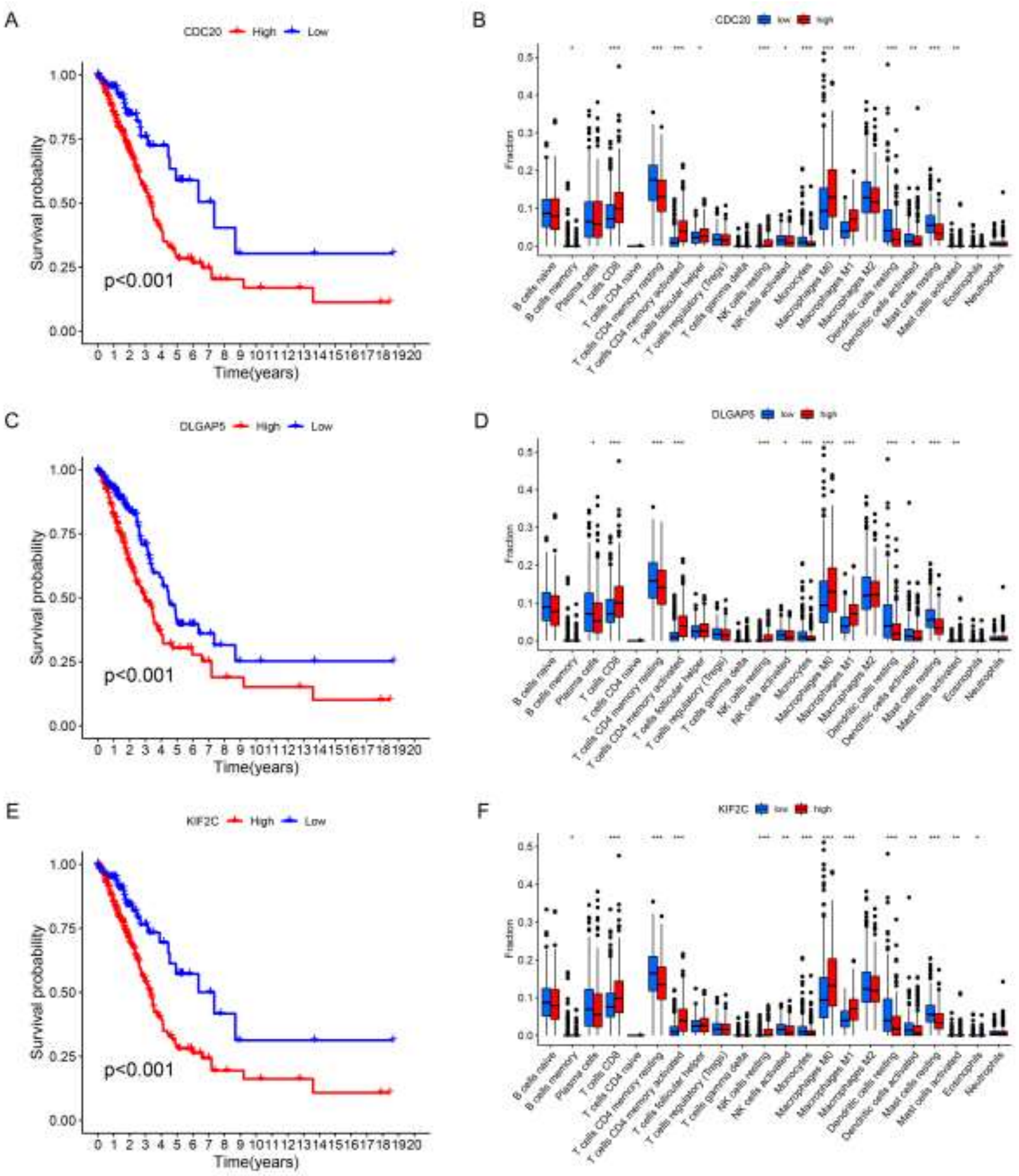

Supplementary Figure 3

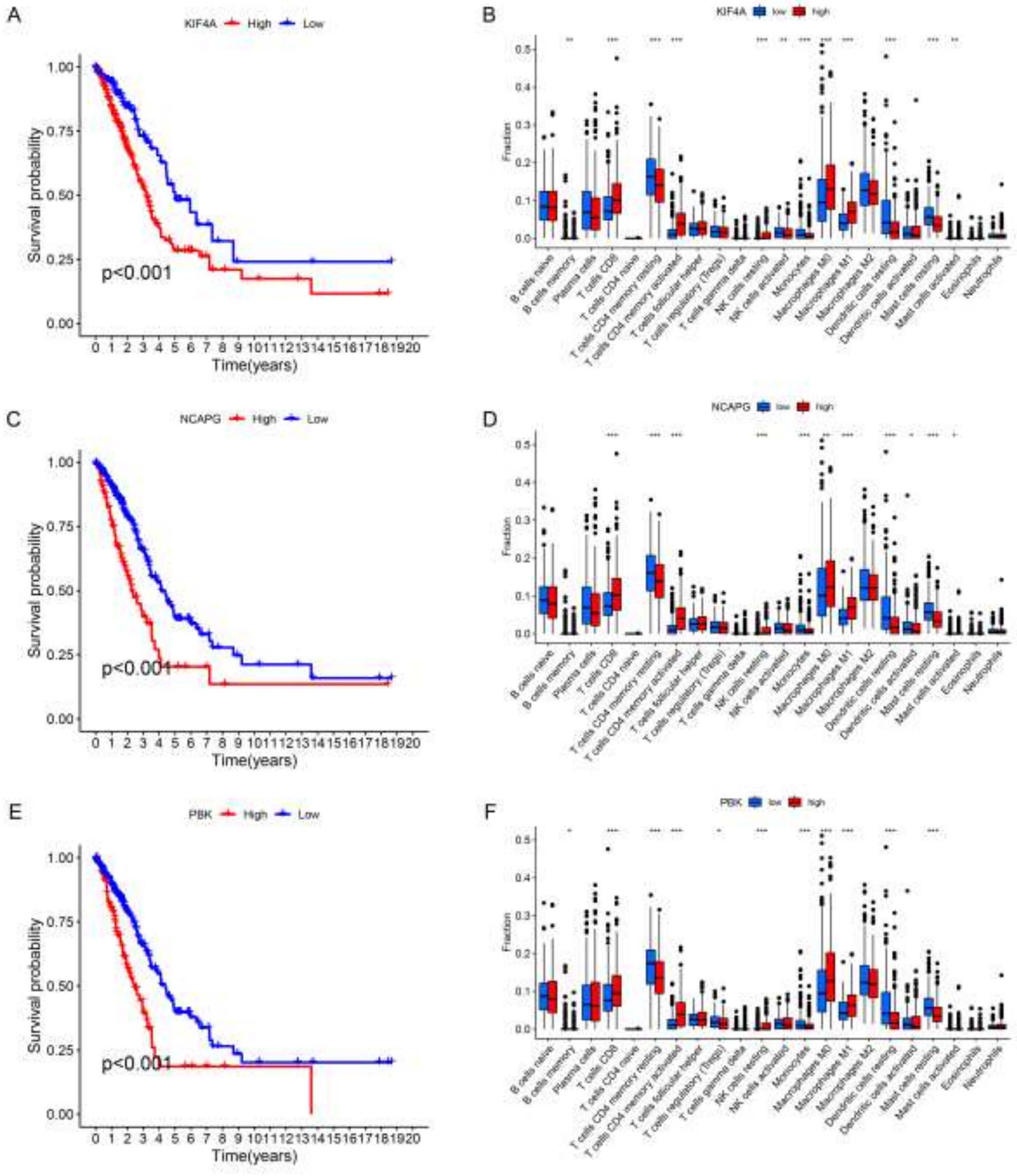

Supplement: Supplementary file 1 [file medi-102-e34844-s001.pdf]
